# Supplementary material for: The interactome of intact mitochondria by cross-linking mass spectrometry provides evidence for coexisting respiratory supercomplexes
Source: Mol Cell Proteomics. 2017 Dec 8;17(2):216–32. doi: 10.1074/mcp.RA117.000470 (PMC5795388; doi:10.1074/mcp.RA117.000470)
Supplement: Supplemental Data [file supp_RA117.000470_133922_0_supp_23978_fzffwf.zip › spectra_annotation/mito_spectra_annotation/14-1-5-1-7-1.pdf]

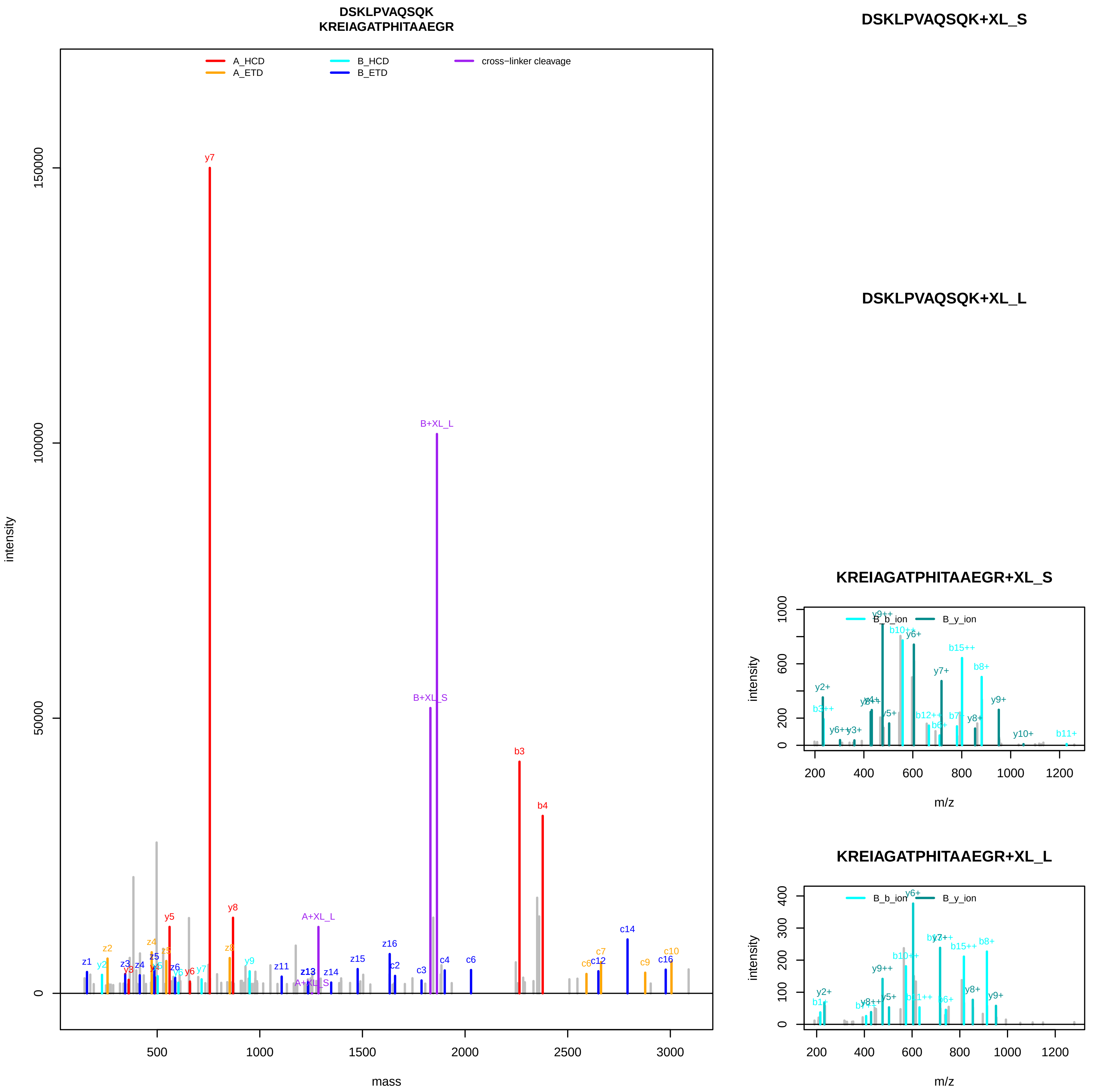
DSKLPVAQSQK
KREIAGATPHITAAEGR
A_HCD
A_ETD
B_HCD
B_ETD
cross−linker cleavage
intensity
mass
0
50000
100000
150000
500
1000
1500
2000
2500
3000
y7
B+XL_L
B+XL_S
b3
b4
y5
y8
A+XL_L
A+XL_S
z1
z2
z3
z4
z5
z6
z8
z11
z13
z14
z15
z16
c2
c3
c4
c6
c7
c9
c10
c12
c14
c16
y2
y3
y4
y6
y9
DSKLPVAQSQK+XL_S
DSKLPVAQSQK+XL_L
KREIAGATPHITAAEGR+XL_S
B_b_ion
B_y_ion
m/z
0
200
600
1000
400
800
1200
y9++
b10++
y6+
b15++
y7+
b8+
y2+
b3++
y9+
y10+
b11+
KREIAGATPHITAAEGR+XL_L
100
300
y8+
y5+
b1+
